# Dose-related and contextual aspects of suboptimal adherence to antiretroviral therapy among persons living with HIV in Western Europe

**DOI:** 10.1093/eurpub/ckaa229

**Published:** 2021-01-18

**Authors:** Babatunde Akinwunmi, Daniel Buchenberger, Jenny Scherzer, Martina Bode, Paolo Rizzini, Fabio Vecchio, Laetitia Roustand, Gaelle Nachbaur, Laurent Finkielsztejn, Vasiliki Chounta, Nicolas Van de Velde

**Affiliations:** 1 Zatum LLC, Department of Epidemiology and Real-World Evidence Grand Blanc, MI, USA; 2 Ipsos Insights LLC, New York, NY, USA; 3 ViiV Healthcare Limited, Munich, Germany; 4 ViiV Healthcare Limited, Verona, Italy; 5 GlaxoSmithKline Pharmaceuticals, Saint Amant les Eaux, France; 6 ViiV Healthcare Limited, Rueil-Malmaison, France; 7 ViiV Healthcare, Brentford Middlesex, UK

## Abstract

**Background:**

The daily oral dosing requirement for antiretroviral therapy (ART) may be challenging for some people living with HIV (PLWHIV) with comorbid conditions, confidentiality concerns or pill fatigue. We investigated suboptimal adherence from the perspective of PLWHIV and HIV physicians.

**Methods:**

PLWHIV on ART (*n* = 688) and HIV physicians (*n* = 120) were surveyed during 2019 in France, Germany, Italy and the UK. Suboptimal adherence was a report the participant missed taking their dose as prescribed ‘Sometimes’/‘Often’/‘Very often’. Physicians’ interest in offering a hypothetical long-acting HIV regimen for suboptimally adherent patients was assessed. Descriptive and multivariable analyses were performed (*P* < 0.05).

**Results:**

Of PLWHIV, 23.8% (164/688) reported suboptimal adherence vs. providers’ estimated prevalence of 33.6% (SD = 28.8). PLWHIV-reported prevalence of specific suboptimal adherence behaviors were: mistimed dose [16.1% (111/688)]; missed a dose [15.7% (108/688)]; dosed under wrong conditions [e.g. food restrictions, 10.5% (72/688)] and overdosed [3.3% (23/688)]. Odds of suboptimal adherence were higher among those with vs. without a report of the following: dysphagia (AOR = 3.61, 95% CI = 2.28–5.74), stress/anxiety because of their daily dosing schedule (AOR = 3.09, 95% CI = 1.97–4.85), gastrointestinal side effects (AOR = 2.09, 95% CI = 1.39–3.15), neurocognitive/mental health conditions (AOR = 1.88, 95% CI = 1.30–2.72) or hiding their HIV medication (AOR = 1.51, 95% CI = 1.04–2.19). Of providers, 84.2% indicated they Definitely/Probably will offer a hypothetical long-acting HIV regimen ‘for patients who have suboptimal levels of adherence to daily oral therapy (50–90%) for non-medical reasons’.

**Conclusions:**

Dysphagia, stressful daily oral dosing schedule, gastrointestinal side effects, neurocognitive/mental health conditions and confidentiality concerns were associated with suboptimal adherence in our study. Adherence support and alternative regimens, such as long-acting antiretroviral therapies, could help address these challenges.

## Introduction

Despite significant progress in Western Europe towards the 90-90-90 targets which aim to increase HIV diagnosis, coverage and viral suppression among people living with HIV (PLWHIV),^1–3^ adherence to daily oral antiretroviral therapy (ART) is still a challenge for some PLWHIV. The increasing life expectancy among PLWHIV on modern ART underscores the need for strategies to encourage adherence.^4–9^ Factors influencing suboptimal adherence are varied and can be at the level of the patient, the drug and the ‘environment’, with the latter comprising both the microenvironment (e.g. drug delivery medium) and the macroenvironment (broader cultural/social environment and health systems).^10–16^

Prevention-wise, factors predisposing to suboptimal adherence may be considered either modifiable or non-modifiable. With recent advances in HIV care, optimal adherence can now be potentially achieved even in the presence of non-modifiable patient factors that lead to poor adherence. For example, non-oral long-acting ARTs administered by healthcare professionals are in development ^17^^,^^18^ which could improve adherence among patients with certain medical conditions that make self-administration of daily oral medicines challenging because of impaired memory or because of problems with swallowing or absorption. Regimen simplicity is key to achieving optimal adherence as complex regimens are neither compatible with normal quality of life (QoL) nor followed exactly as prescribed.^19^^,^^20^

The European AIDS Clinical Society guidelines emphasize the importance of regular assessment of barriers faced by PLWHIV to ensure tailored care to meet patients’ needs and preferences.^21^ Data on contextual aspects of suboptimal adherence (e.g. dosing conditions such as with food, or timing of doses) are especially needed because of their potential impact on the drug’s bioavailability and effectiveness; previous research has largely focused on quantifying the number or proportion of missed doses.^22^ To provide a comprehensive assessment of suboptimal ART adherence, the aims of this study were to: (i) investigate prevalence of suboptimal adherence from the perspective of both PLWHIV and healthcare providers (HCPs). (ii) Examine the percentage of PLWHIV reporting various subtypes of suboptimal adherent behaviors in relation to the frequency, timing, dosage, and conditions under which ART is taken. (iii) Explore factors associated with receptivity towards pharmacologic interventions with the potential to improve adherence.

## Methods

### Study population/sampling approach

In 2019, we conducted a web-based, anonymous survey of 698 PLWHIV in France, Germany, Italy and the UK. Of these, 688 were currently on ART and comprise the analytic sample; those not on ART (*n* = 10) were excluded. The following inclusion criteria were used to recruit participants: (i) aged ≥18 years, any gender; (ii) confirmed HIV status with a photograph of their HIV medication/prescription with their name on it. Approximately 60–70% of PLWHIV were recruited from existing panels of confirmed HIV sero-positive individuals; the remainder were recruited from various charities/support groups, online support programs/communities and social media platforms. Ipsos Healthcare monitored PLWHIV recruitment on a weekly basis to ensure that the recruited sample’s composition aligned with the national HIV population on key characteristics (age, gender, sexual orientation and country of origin). Detailed recruitment of participants has been published elsewhere.^23^ Overall response rate was 64.3%.

A non-probability sample of 120 physicians was recruited (30 per country). Inclusion criteria were: (i) board certified/eligible physician with ≥5 years of practice as an internist or HIV/infectious disease specialist; (ii) personally managed ≥50 unique HIV patients and saw ≥15 weekly. All participants were compensated for their participation.

### Ethical review

Ethical review and approval for this study was by the Pearl Institutional Review Board (study number 19-IPSO-125).

### Measures

#### Past-month frequency, specific domains and reasons for suboptimal adherence

**Figure 1:**
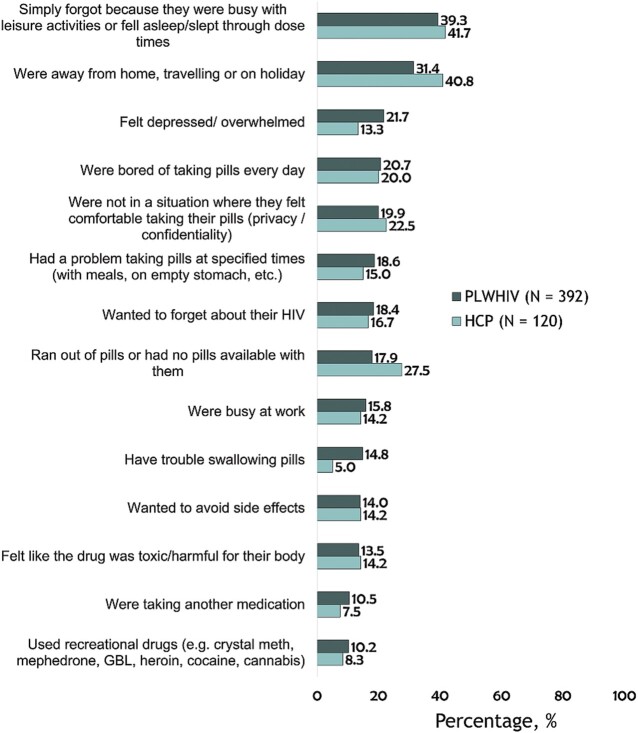
Comparison of reasons perceived by HCPs (*n* = 120) for their patients missing ART ‘Often’ vs. PLWHIV-reported reasons for missing ART among those with any level of suboptimal adherence (*n* = 392). *Note:* ART, antiretroviral therapy; PLWHIV, people living with HIV; HCPs, healthcare providers. Respondents were classified as reporting any level of suboptimal adherence if they provided a response of ‘Rarely’, ‘Sometimes’, ‘Often’, or ‘Very Often’ to the question: ‘When we consider adherence to treatment, not only in terms of missed doses but also taking the pills at the right time and under the right conditions without overdosing, in the past month how often have you missed taking your HIV pills exactly as prescribed by your HIV physician?’ For each of the reasons listed, PLWHIV who reported missing ART for that reason ‘Sometimes’, ‘Often’, or ‘Very Often’ were classified as positive responses. The percentage reported by HCPs are estimates of what percentage of their patients experience the assessed issue ‘Often’

The following three adherence-related questions were asked, each measured on an ordinal scale of 1—‘Never’, 2—‘Rarely’, 3—‘Sometimes’, 4—‘Often’ or 5—‘Very often’. Unless otherwise specified, all constructs reported are from the PLWHIV survey.

(Q1) ‘*When we consider adherence to treatment, not only in terms of missed doses but also taking the pills at the right time and under the right conditions without overdosing, in the past month how often have you missed taking your HIV pills exactly as prescribed by your HIV physician?*’ Participants answering ≥3 (‘Sometimes’/‘Often’/‘Very often’) were classified as having some level of suboptimal adherence. We also explored a more sensitive definition encompassing scores ≥2 (i.e. ‘Rarely’/‘Sometimes’/‘Often’/‘Very often’). We used the more specific definition (≥‘Sometimes’) within overall analyses among all participants. The more sensitive definition (≥‘Rarely’) was used within subgroup analyses to ensure adequate sample size in analyses restricted to those reporting any suboptimal adherence behavior, regardless of frequency. All subsequent appearances of ‘suboptimal adherence’ in this article specifically refer to the former definition (i.e. missing ART ≥ ‘Sometimes’) whereas all instances of the latter definition are explicitly framed as missing ART ≥ ‘Rarely’. Physicians were asked: ‘what percentage of your patients on ART do you believe are not perfectly adhering to their regimen?’ Possible responses range from 0 to 100% (analyzed as means).

(Q2) ‘*When you missed taking your HIV pills exactly as prescribed, in the past month how frequent were the following reasons?*’ Four specific suboptimal adherence behaviors were then assessed, each on a separate row with its own response options: *(a)* ‘*Missed one dose*’*; (b)* *‘Dose not taken at the right time*’*; (c)* *‘Dose not taken under the right conditions (*e.g. *with meals, on empty stomach)’; or (d)* *‘Overdosing* (i.e. *taking HIV medication twice because you were not sure)*’. PLWHIV who reported some level of suboptimal adherence (Q1 above) *and* answered ≥3 to the specified behavior in Q2 (a–d, respectively) were classified as reporting some level of the specific suboptimal adherence behavior. A similar question was asked in the HCP survey: ‘When your patients miss taking their HIV pills exactly as prescribed, how common are each of the following reasons?’ Response options were on a 5-point scale [1—Never, 2—Rarely, 3—Sometimes, 4—Often, 5—Very Often]. Scores of ≥4 were classified as presence of the perception that suboptimal adherence for the specific behavior was prevalent among their patients.

(Q3) ‘*Besides the specific medical conditions above, people may miss taking their HIV medications for various reasons. In the past month, how often have you missed taking your HIV medications because you…*’ The specific reasons assessed are listed in figure 1. For each assessed reason, PLWHIV with scores ≥3 were classified as having missed ART for the specified reason. These same reasons were assessed in the HCP survey but measured on a 4-point scale [1—Never, 2—Rarely, 3—Sometimes, 4—Often]. A score of 4 by HCP participants was classified as presence of the perception that the specified reason for missing ART was prevalent among their patients.

#### Perceptions towards long-acting HIV treatment and its potential impact on improving adherence

The PLWHIV survey assessed participants’ perceptions towards a novel long-acting regimen of cabotegravir (CAB) and rilpivirine (RPV) (CAB + RPV LA), a new treatment that has been shown to be well-tolerated and as efficacious as daily oral ART regimens in maintaining virologic suppression in phase III randomized clinical trials.^17^^,^^18^ Within the survey, relevant facts (from clinical trials) as well as certain assumptions were provided about the new treatment (described as a hypothetical ‘Regimen Z’) to orient the respondents, including information on the route (‘injectable regimen’), frequency (‘every two months’), settings (‘given by a nurse, doctor, or other healthcare professional’), effectiveness (‘as effective at keeping patients undetectable as other HIV treatments’), possible injection site reactions (soreness, which ‘typically lasts 3 days or less’) and assumed cost (‘at similar cost to you compared with your current treatment’).

Participants in the PLWHIV survey who reported missing ART ≥ ‘Rarely’ were asked—‘Previously, you mentioned that you missed taking your HIV pills exactly as prescribed by your HIV physician due to various reasons. Do you think that, overall, Regimen Z would help to improve your current level of adherence?’ A response of ‘Yes’ was classified as an affirmative answer whereas responses of ‘No’ or ‘Not sure’ were classified as absence of an affirmative answer.

Within the HCP survey, physicians were asked their perceptions on the relative impact of various other medical conditions on suboptimal adherence. They were also asked ‘how likely [they] would be to offer Regimen Z to those patients… who have suboptimal levels of adherence to daily oral therapy (50–90%) for non-medical reasons (e.g. travels, age/maturity, work, recreational drug use)’. Response options were on a 4-point scale [1—Definitely Will Offer, 2—Probably Will Offer, 3—Probably Won’t Offer, 4—Definitely Won’t Offer].

### Analyses

The proportion reporting suboptimal adherence and its subtypes were calculated among all PLWHIV on ART in the four countries combined (*n* = 688). Subgroup differences were assessed with chi-squared tests or ANOVA, as appropriate, at *P* < 0.05.

Exploratory multivariable logistic regression analyses were used to explore sociodemographic and clinical factors associated with: (i) reporting suboptimal adherence (among all PLWHIV on ART, *n* = 688). (ii) Specific reasons for missing medications (among PLWHIV on ART who reported missing ART ≥ ‘Rarely’, *n* = 392). (iii) perception that the hypothetical long-acting regimen might help improve adherence (among PLWHIV on ART who reported missing ART ≥ ‘Rarely’ and responded to the question on the long-acting regimen, *n* = 374). All analyses were conducted using R Version 3.6.1.

## Results

Of surveyed PLWHIV on ART, 68.3% were employed, 66.4% were men, 60.6% homosexual and 78.6% had a college degree or higher. HCPs estimated that 85.7% of their patients were currently on ART.

### Prevalence of suboptimal adherence and its subtypes

Overall, 23.8% (164/688) of surveyed PLWHIV reported suboptimal adherence in general. Specific subtypes of suboptimal adherence behaviors reported were: mistimed dose [16.1% (111/688)]; missed a dose [15.7% (108/688)]; dosed under wrong conditions [10.5% (72/688)]; and overdosed [3.3% (23/688)]. Surveyed HCPs estimated that 33.6% (SD = 28.8) of their patients had suboptimal adherence.

### Factors associated with suboptimal adherence

Within bivariate analyses, suboptimal adherence was significantly higher among PLWHIV who reported hiding medications, on multi-tablet regimens, women and those with longer HIV duration, but did not vary by type of ART regimen (e.g. integrase strand transfer inhibitors, nucleoside or non-nucleoside reverse transcriptase inhibitors or protease inhibitors) (table 1). The percentage reporting suboptimal adherence however differed by presence or absence, respectively, of self-reported difficulty swallowing [46.3% (57/123) vs. 18.9% (107/565), *P* < 0.001] or neurocognitive/mental health conditions [32.4% (79/244) vs. 19.1% (85/444), *P* < 0.001].

**Table 1:** Characteristics of surveyed people living with HIV and prevalence of reported suboptimal adherence behaviors among all participants on ART (*n* = 688)

	Tabulation variables		Subtypes of suboptimal adherence behaviors				Aggregate
Characteristics	Levels	% (*n*)	Dosed at wrong time, %	Missed a dose	Dosed under wrong conditions, %	Overdosed, %	Some level of suboptimal adherence, %
Total	**Overall**	**100.0 (688)**	16.1	15.7	10.5	3.3	23.8
Country	France	20.9 (144)	13.2	**23.6**	13.2	**7.6**	27.1
	Germany	28.8 (198)	15.2	**11.1**	9.6	**1.5**	21.7
	Italy	21.8 (150)	16.7	**12.7**	8.0	**2.0**	20.7
	UK	28.5 (196)	18.9	**16.8**	11.2	**3.1**	26.0
Year of diagnosis	2017–19	12.8 (88)	8.0	8.0	6.8	2.3	**10.2**
	2010–16	41.6 (286)	17.5	16.4	11.9	4.5	**25.5**
	Pre-2010	45.6 (314)	17.2	17.2	10.2	2.5	**26.1**
Age, years	<50	70.4 (484)	15.7	16.3	11.2	4.1	24.0
	50+	29.7 (204)	17.2	14.2	8.8	1.5	23.5
Gender	Men	66.4 (457)	**13.1**	**12.9**	**7.7**	**2.0**	**20.8**
	Women	33.3 (229)	**22.3**	**21.4**	**16.2**	**6.1**	**30.1**
	Other	0.3 (2)	—	—	—	—	—^f^
Sexual orientation	Heterosexual	33.9 (233)	**20.2**	**19.3**	**13.7**	4.7	27.5
	Homosexual	60.6 (417)	**12.7**	**12.9**	**7.9**	2.2	20.9
	Other	5.5 (38)	**28.9**	**23.7**	**18.4**	7.9	34.2
Gender/Sexual orientation	Men who have sex with men	59.2 (407)	**12.5**	**13.0**	**7.4**	**2.0**	20.6
	Men who have sex with women	4.4 (30)	**20.0**	**13.3**	**10.0**	**3.3**	23.3
	Women	33.3 (229)	**22.3**	**21.4**	**16.2**	**6.1**	30.1
	Other/unknown	3.2 (22)	**13.6**	**9.1**	**9.1**	**0.0**	18.2
Nativity status	Foreign-born	37.7 (259)	**20.1**	**22.4**	**15.1**	**6.2**	**29.3**
	Native-born	62.4 (429)	**13.8**	**11.7**	**7.7**	**1.6**	**20.5**
Marital status	Single	40.8 (273)	**12.1**	15.0	**7.7**	1.5	**20.9**
	Married/with partner	52.5 (351)	**17.4**	14.8	**10.8**	4.8	**24.2**
	Widowed/divorced/ separated	6.7 (45)	**26.7**	26.7	**20.0**	4.4	**37.8**
Education	Post-graduate	20.0 (134)	14.2	13.4	11.2	6.7	20.9
	College	58.6 (392)	14.8	16.3	8.9	2.0	24.0
	General Certificate of Secondary Education	14.8 (99)	22.2	16.2	14.1	5.1	29.3
	Other	6.6 (44)	15.9	15.9	9.1	2.3	18.2
Employment status	Employed	68.3 (457)	15.3	14.7	9.0	3.7	23.4
	Non-employed	31.7 (212)	17.0	17.9	12.7	2.8	24.5
Domicile	Metropolitan area	69.5 (465)	15.7	15.7	9.7	4.1	22.8
	Nonmetropolitan area	30.5 (204)	16.2	15.7	11.3	2.0	26.0
ART Formulation	Single-tablet regimen	55.4 (381)	**13.4**	**12.3**	9.2	3.4	**20.7**
	Multi-tablet regimen	44.6 (307)	**19.5**	**19.9**	12.1	3.3	**27.7**
NNRTI as core agent^a^	No	65.4 (450)	16.4	16.4	11.3	3.3	24.7
	Yes	34.6 (238)	15.5	14.3	8.8	3.4	22.3
INSTI as core agent^b^	No	43.6 (300)	16.0	14.7	9.7	2.3	22.0
	Yes	56.4 (388)	16.2	16.5	11.1	4.1	25.3
Protease inhibitor as core agent^c^	No	78.2 (538)	**14.3**	**13.8**	**8.6**	**2.4**	22.3
	Yes	21.8 (150)	**22.7**	**22.7**	**17.3**	**6.7**	29.3
Entry inhibitor as core agent^d^	No	96.1 (661)	15.7	15.4	**9.8**	**2.9**	23.3
	Yes	3.9 (27)	25.9	22.2	**25.9**	**14.8**	37.0
Emotional rating of ART experience^e^	Positive	52.0 (358)	14.8	**12.6**	10.9	**5.3**	**20.1**
	Negative	48.0 (330)	17.6	**19.1**	10.0	**1.2**	**27.9**
Ever hidden or disguised HIV medication in past 6 months	No	56.7 (390)	**13.1**	**12.6**	9.0	**2.1**	**20.8**
	Yes	43.3 (298)	**20.1**	**19.8**	12.4	**5.0**	**27.9**

*Note:* ART, antiretroviral therapy; NNRTI, non-nucleoside reverse transcriptase inhibitor; INSTI, integrase strand transfer inhibitor. Classes of ART are not mutually exclusive. Results in bold indicate statistically significant group differences based on χ^2^ tests (*P* < 0.05).

a NNRTI-containing regimens included ‘Atripla^®^ or generics (emtricitabine/efavirenz/tenofovir disoproxil fumarate)’; ‘Delstrigo (doravirine/lamivudine/tenofovir disoproxil fumarate)’; ‘Edurant (rilpivirine)’; ‘Eviplera (emtricitabine/rilpivirine/tenofovir-disoproxil fumarate)’; ‘Viramune or generics (Nevirapin)’; ‘Sustiva or generics (efavirenz)’; ‘Odefsey (emtricitabine/rilpivirine/tenofovir alafenamide)’ or ‘Pifeltro (doravirine)’.

b INSTI-containing regimens included ‘Genvoya (elvitegravir/cobicistat/emtricitabine/tenofovir alafenamide)’; ‘Tivicay (dolutegravir)’; ‘Triumeq (dolutegravir/abacavir/lamivudine)’; ‘Isentress (raltegravir)’; ‘Juluca (dolutegravir/rilpivirine)’; ‘Stribild (elvitegravir/cobicistat/emtricitabine/tenofovir disoproxil fumarate)’ or ‘Biktarvy (bictegravir/emtricitabine/tenofovir alafenamide)’.

c Protease Inhibitor-containing regimens included ‘Kaletra (lopinavir/ritonavir)’; ‘Evotaz (atazanavir/cobicistat)’; ‘Prezista (darunavir)’; ‘Reyataz (atazanavir)’; ‘Rezolsta (darunavir/cobicistat)’; or ‘Symtuza (darunavir/emtricitabine/tenofovir alafenamide)’.

d Entry Inhibitor-containing regimens included ‘Celsentri (maraviroc)’; ‘Fuzeon (enfuvirtide)’ or ‘Fostemsavir’.

e Individuals with ratings of ≤0 on a scale measuring emotional experience with their HIV medication that ranged from −50 to +50 (negative numbers indicate perceived negative experiences) were classified as having a negative emotional experience with their HIV medicines.

f Results not presented because of small sample size.

Within multivariable logistic regression analyses among all surveyed PLWHIV on ART (*n* = 688), higher odds of suboptimal adherence were seen among those reporting vs. not reporting: gastrointestinal ART side effects (i.e. ‘stomach/gastric problems because of the medication’) (AOR = 2.09, 95% CI = 1.39–3.15), neurocognitive/mental health conditions (AOR = 1.88, 95% CI = 1.30–2.72), dysphagia (AOR = 3.61, 95% CI = 2.28–5.74), hiding their HIV medication (AOR = 1.51, 95% CI = 1.04–2.19) and that their daily dosing schedule caused them stress (AOR = 3.09, 95% CI = 1.97–4.85). Furthermore, higher odds of suboptimal adherence were seen among women vs. men (AOR = 1.97, 95% CI = 1.34–2.88), and among those diagnosed with HIV during 2010–16 (AOR = 3.68, 95% CI = 1.71–7.90), or pre-2010 (AOR = 3.72, 95% CI = 1.75–7.94) vs. 2017–19 (table 2).

**Table 2:** Adjusted logistic regression analysis for factors associated with suboptimal ART adherence among all surveyed people living with HIV on ART as well as predisposing factors for receptivity towards long-acting HIV treatment to improve adherence among participants who reported any level of suboptimal adherence

Characteristics	Categories	Among all participants on ART	Among participants on ART who reported any level of suboptimal adherence to ART ^g^
		Factors associated with some level of suboptimal adherence^f^ (688)	Factors associated with perception a long-acting regimen will help them (374)
Age	50+ vs. <50 years	0.84 (0.54–1.31)	0.51 (0.28–0.94)*
Education	College vs. postgraduate	1.26 (0.77–2.06)	2.65 (1.28–5.46)*
	Secondary vs. postgraduate	1.75 (0.90–3.41)	2.11 (0.77–5.80)
Employment status	Non-employed vs. employed	0.95 (0.63–1.44)	0.81 (0.44–1.50)
Domicile	Non-metropolitan vs. metropolitan	1.07 (0.72–1.59)	0.96 (0.51–1.81)
Regimen formulation	Multi-tablet regimen vs. single-tablet regimen	1.22 (0.84–1.78)	0.78 (0.44–1.37)
	NNRT-containing regime (yes vs. no)^a^	0.78 (0.53–1.15)	0.77 (0.44–1.36)
	INSTI-containing regimen (yes vs. nz)^b^	1.18 (0.81–1.72)	1.43 (0.82–2.48)
	PI-containing regimen (yes vs. no)^c^	1.39 (0.90–2.14)	1.29 (0.64–2.59)
	EI-containing regimen (yes vs. no)^d^	1.78 (0.77–4.15)	1.07 (0.27–4.27)
Side effects^e^	Gastrointestinal vs. none	2.09 (1.39–3.15)*	2.27 (1.23–4.22)*
	Non-gastrointestinal only vs. none	1.42 (0.81–2.46)	2.32 (0.98–5.50)
Emotional challenges	Perception daily ART dosing schedule is stressful (yes vs. no)	3.09 (1.97–4.85)*	4.60 (1.58–13.35)*
Confidentiality concerns	Reported hiding/disguising ART (yes vs. no)	1.51 (1.04–2.19)*	1.26 (0.72–2.19)
Medical conditions	Neurocognitive/mental health conditions (yes vs. no)	1.88 (1.30–2.72)*	1.93 (1.08–3.45)*
	Gastrointestinal conditions interfering with oral intake (yes vs. no)	1.32 (0.83–2.08)	2.26 (1.00–5.08)*
	Dysphagia (yes vs. no)	3.61 (2.28–5.74)*	2.97 (1.26–7.01)*
	Malabsorption (yes vs. no)	1.14 (0.61–2.13)	1.04 (0.42–2.58)
Year of HIV diagnosis	2010–16 vs. 2017–19	3.68 (1.71–7.90)*	1.05 (0.28–3.91)
	Pre-2010 vs. 2017–19	3.72 (1.75–7.94)*	0.35 (0.10–1.24)
Country	Germany vs. France	0.83 (0.50–1.40)	0.60 (0.26–1.35)
	Italy vs. France	0.71 (0.41–1.23)	0.39 (0.17–0.89)*
	UK vs. France	1.01 (0.61–1.69)	0.74 (0.32–1.68)
Gender	Women vs. men	1.97 (1.34–2.88)*	1.43 (0.79–2.59)

*Note:* NA, not applicable. Each independent variable was analyzed separately, adjusting for country, gender, and duration of disease. Asterisks (*) indicate statistically significant results at *P* < 0.05. ART, antiretroviral therapy; NNRTI, non-nucleoside reverse transcriptase inhibitor; INSTI, integrase strand transfer inhibitor; PI, protease inhibitor; EI, entry inhibitor. Classes of ART are not mutually exclusive.

a NNRTI-containing regimens included ‘Atripla^®^ or generics (emtricitabine/efavirenz/tenofovir disoproxil fumarate)’; ‘Delstrigo (doravirine/lamivudine/tenofovir disoproxil fumarate)’; ‘Edurant (rilpivirine)’; ‘Eviplera (emtricitabine/rilpivirine/tenofovir -disoproxil fumarate)’; ‘Viramune or generics (Nevirapin)’; ‘Sustiva or generics (efavirenz)’; ‘Odefsey (emtricitabine/rilpivirine/tenofovir alafenamide)’; or ‘Pifeltro (doravirine)’.

b INSTI-containing regimens included ‘Genvoya (elvitegravir/cobicistat/emtricitabine/tenofovir alafenamide)’; ‘Tivicay (dolutegravir)’; ‘Triumeq (dolutegravir/abacavir/lamivudine)’; ‘Isentress (raltegravir)’; ‘Juluca (dolutegravir/rilpivirine)’; ‘Stribild (elvitegravir/cobicistat/emtricitabine/tenofovir disoproxil fumarate)’; or ‘Biktarvy (bictegravir/emtricitabine/tenofovir alafenamide)’.

c Protease inhibitor-containing regimens included ‘Kaletra (lopinavir/ritonavir)’; ‘Evotaz (atazanavir/cobicistat)’; ‘Prezista (darunavir)’; ‘Reyataz (atazanavir)’; ‘Rezolsta (darunavir/cobicistat)’; or ‘Symtuza (darunavir/emtricitabine/tenofovir alafenamide)’.

d Entry inhibitor-containing regimens included ‘Celsentri (maraviroc)’; ‘Fuzeon (enfuvirtide)’ or ‘Fostemsavir’.

e A history of a major side effect was said to be present if the respondent reported a past adverse effect from HIV medication (e.g. ‘stomach/gastric problems because of the medication’ or ‘difficulties taking my HIV treatment as I was having too many side effects’), that led to stopping ART, switching ART or failing to achieve viral suppression from non-adherence.

f Respondents were classified as reporting some level of suboptimal adherence if they provided a response of ‘Sometimes’, ‘Often’, or ‘Very Often’ to the question: ‘When we consider adherence to treatment, not only in terms of missed doses but also taking the pills at the right time and under the right conditions without overdosing, in the past month how often have you missed taking your HIV pills exactly as prescribed by your HIV physician?’.

g Respondents were classified as reporting any level of suboptimal adherence if they provided a response of ‘Rarely’, ‘Sometimes’, ‘Often’, or ‘Very Often’ to the question: ‘When we consider adherence to treatment, not only in terms of missed doses but also taking the pills at the right time and under the right conditions without overdosing, in the past month how often have you missed taking your HIV pills exactly as prescribed by your HIV physician?’.

Top reasons for missing ART doses among those who reported missing ART ≥ ‘Rarely’ included being busy with leisure activities or fell asleep/slept through dose time [39.3% (154/392)], away from home, travelling or on holiday [31.4% (123/392)] and feeling depressed/overwhelmed [21.7% (85/392)] (figure 1). In multivariable analyses restricted to those reporting missing ART ≥ ‘Rarely’, participants who felt stressed by their daily dosing schedule had 2-fold higher odds of missing ART for the following reasons compared with those not feeling stressed by their daily dosing schedule: wanted to avoid side effects (AOR = 2.48), felt depressed/overwhelmed (AOR = 2.12), were away from home (AOR = 2.21), busy with leisure activities (AOR = 2.01), bored of taking pills every day (AOR = 2.24), busy at work (AOR = 2.28) and wanted to forget about HIV (AOR = 2.25) (all *P* < 0.05, table 3). Women were more likely than men to miss ART because of feeling depressed/overwhelmed (AOR = 3.17, 95% CI = 1.60–6.29), whereas no gender differences existed in missing ART because of being busy or because of travel. Compared with those not reporting side effects from their HIV medication, a report of gastrointestinal ART side effects increased the odds of missing ART because of feeling depressed/overwhelmed (AOR =1.98, 95% CI = 1.01–3.88), and to avoid side effects (AOR = 3.68, 1.25–10.83). Other significant correlates are shown in table 3.

**Table 3:** Adjusted logistic regression analysis for factors associated with report of specific reasons for missing medications among people living with HIV who reported any level of suboptimal adherence to ART^a^, 2019 (*n* = 392)

Characteristics	Medical reasons						Non-medical reasons							
	Wanted to avoid side effects	Perceived the drug as toxic	Felt depressed/ overwhelmed	Problem taking pills with meals/ empty stomach	Have trouble swallowing pills	Were taking another medication	Away from home, travelling or on holiday	Busy with leisure activities or slept through dose time	Was in a situation where you had privacy concerns	Ran out of pills/had no pills	Bored of taking pills every day	Busy at work	Wanted to forget about HIV	Were using recreational drugs
Age														
50+ vs. <50 years	0.73	2.35	1.48	1.44	1.73	1.81	0.77	1.28	0.94	0.45	1.20	0.74	0.64	0.59
	(0.24–2.17)	(0.89–6.15)	(0.71–3.10)	(0.69–3.03)	(0.63–4.75)	(0.59–5.53)	(0.41–1.42)	(0.72–2.26)	(0.40–2.19)	(0.20–1.02)	(0.55–2.61)	(0.32–1.73)	(0.27–1.53)	(0.21–1.63)
Education														
College vs. postgraduate	0.51	1.12	1.16	0.94	1.48	0.91	0.93	1.57	1.07	0.82	1.15	1.22	0.92	0.75
	(0.20–1.28)	(0.47–2.66)	(0.55–2.44)	(0.45–1.99)	(0.59–3.69)	(0.37–2.26)	(0.50–1.73)	(0.85–2.92)	(0.50–2.28)	(0.39–1.73)	(0.53–2.48)	(0.56–2.66)	(0.42–1.99)	(0.32–1.80)
GCSE vs. postgraduate	0.66	0.93	0.79	0.77	1.67	0.19	1.13	1.65	1.66	1.48	1.37	0.91	1.66	1.17
	(0.16–2.71)	(0.24–3.66)	(0.26–2.38)	(0.27–2.19)	(0.44–6.32)	(0.02–1.80)	(0.49–2.60)	(0.72–3.78)	(0.55–5.02)	(0.47–4.67)	(0.45–4.11)	(0.29–2.84)	(0.51–5.38)	(0.34–4.03)
Other vs. postgraduate	0.76	1.67	0.95	0.41	0.89	1.31	0.37	0.90	0.65	1.25	1.20	1.07	1.47	2.29
	(0.15–3.74)	(0.40–6.98)	(0.28–3.21)	(0.09–1.80)	(0.17–4.60)	(0.26–6.76)	(0.11–1.31)	(0.32–2.54)	(0.14–3.08)	(0.35–4.49)	(0.33–4.33)	(0.27–4.29)	(0.40–5.41)	(0.61–8.63)
ART formulation														
Multi- vs. single table regimen	0.72	0.51	0.90	0.69	0.89	1.33	1.07	1.19	1.18	0.69	1.02	0.96	1.03	1.30
	(0.31–1.68)	(0.23–1.13)	(0.48–1.67)	(0.36–1.30)	(0.40–2.02)	(0.57–3.08)	(0.64–1.78)	(0.74–1.93)	(0.60–2.33)	(0.36–1.33)	(0.53–1.97)	(0.49–1.87)	(0.51–2.06)	(0.61–2.76)
ART side effects^b^														
Gastrointestinal	3.68	2.15	1.98	1.57	7.51	7.79	1.10	1.21	4.31	1.21	3.86	3.00	3.09	0.80
	(1.25–10.83)*	(0.90–5.11)	(1.01–3.88)*	(0.82–3.04)	(2.69–20.98)*	(2.14–28.35)*	(0.64–1.89)	(0.72–2.02)	(1.96–9.50)*	(0.60–2.46)	(1.86–8.00)*	(1.42–6.33)*	(1.41–6.80)*	(0.35–1.82)
Non-gastrointestinal only	1.96	0.49	0.81	0.60	0.38	4.38	1.08	0.94	2.11	1.33	1.07	1.03	1.52	0.72
	(0.46–8.30)	(0.11–2.10)	(0.31–2.09)	(0.21–1.67)	(0.04–3.48)	(0.92–20.81)	(0.52–2.24)	(0.47–1.86)	(0.73–6.10)	(0.50–3.52)	(0.36–3.18)	(0.33–3.18)	(0.51–4.49)	(0.22–2.36)
Perception ART dosing schedule causes stress/anxiety														
Yes vs. no	2.48	1.71	2.12	1.87	2.17	1.04	2.21	2.01	1.82	1.82	2.24	2.28	2.25	1.16
	(1.09–5.63) *	(0.78–3.74)	(1.08–4.15)*	(0.97–3.62)	(0.98–4.79)	(0.43–2.52)	(1.25–3.90)*	(1.14–3.54)*	(0.92–3.59)	(0.91–3.66)	(1.13–4.42)*	(1.16–4.46)*	(1.12–4.51)*	(0.51–2.65)
Hiding HIV medications														
Yes vs. no	0.78	1.83	0.82	0.83	1.23	1.18	1.31	0.95	2.37	0.99	0.94	0.87	1.50	1.46
	(0.34–1.82)	(0.85–3.93)	(0.44–1.51)	(0.45–1.54)	(0.56–2.73)	(0.50–2.79)	(0.79–2.16)	(0.59–1.54)	(1.20–4.67)*	(0.52–1.88)	(0.49–1.79)	(0.45–1.67)	(0.76–2.95)	(0.68–3.12)
Medical condition making oral dosing challenging^c^														
Yes vs. no	5.08	5.26	5.50	2.19	4.28	3.08	1.99	2.51	2.37	0.98	3.10	1.89	4.77	3.53
	(1.75–14.76)*	(1.90–4.53)*	(2.51–12.04)*	(1.11–4.32)*	(1.63–11.22)*	(1.08–8.83)*	(1.18–3.37)*	(1.53–4.12)*	(1.14–4.93)*	(0.51–1.88)	(1.48–6.52)*	(0.94–3.81)	(1.99–11.43)*	(1.31–9.47)*
Year of HIV diagnosis														
2010–16 vs. 2017–19	0.86	0.93	0.41	0.38	0.70	0.47	0.71	0.92	0.80	2.05	0.72	0.65	0.64	0.63
	(0.22–3.33)	(0.24–3.55)	(0.13–1.27)	(0.13–1.07)	(0.18–2.70)	(0.13–1.73)	(0.27–1.84)	(0.35–2.42)	(0.26–2.47)	(0.60–6.95)	(0.22–2.36)	(0.21–1.98)	(0.19–2.10)	(0.18–2.21)
Pre-2010 vs. 2017–19	0.60	0.52	0.74	0.34	0.46	0.25	0.71	1.00	0.59	3.07	1.05	0.50	1.30	0.60
	(0.13–2.72)	(0.12–2.38)	(0.22–2.47)	(0.11–1.07)	(0.10–2.09)	(0.05–1.14)	(0.26–1.98)	(0.36–2.79)	(0.17–2.06)	(0.83–11.31)	(0.29–3.72)	(0.15–1.75)	(0.36–4.66)	(0.15–2.40)
Country														
Germany vs. France	0.10	0.28	0.26	0.71	0.25	0.11	1.02	0.85	0.30	0.24	0.34	1.08	0.11	0.55
	(0.02–0.56)*	(0.07–1.03)	(0.09–0.75)*	(0.30–1.71)	(0.07–0.85)*	(0.01–0.91)*	(0.50–2.10)	(0.42–1.73)	(0.11–0.83)*	(0.09–0.61)*	(0.13–0.88)	(0.40–2.90)	(0.03–0.37)*	(0.18–1.65)
Italy vs. France	0.85	0.88	0.99	0.41	0.49	0.59	1.01	1.55	0.92	1.13	0.75	2.04	0.82	0.76
	(0.33–2.18)	(0.36–2.17)	(0.46–2.15)	(0.17–0.99)*	(0.19–1.27)	(0.23–1.52)	(0.50–2.01)	(0.80–3.03)	(0.41–2.07)	(0.55–2.32)	(0.34–1.65)	(0.88–4.76)	(0.37–1.83)	(0.30–1.97)
UK vs. France	0.95	1.48	2.16	1.43	0.77	0.79	0.81	1.54	0.54	0.15	0.58	1.79	0.56	0.65
	(0.32–2.85)	(0.53–4.12)	(0.97–4.79)	(0.63–3.22)	(0.27–2.19)	(0.27–2.28)	(0.40–1.64)	(0.80–2.99)	(0.22–1.31)	(0.06–0.42)*	(0.25–1.37)	(0.71–4.49)	(0.23–1.36)	(0.23–1.78)
Gender														
Women vs. men	8.97	7.84	3.17	3.52	4.51	2.81	1.61	1.64	2.66	2.25	2.98	1.68	2.48	1.21
	(3.50–22.97)*	(3.30–8.60)*	(1.60–6.29)*	(1.80–6.89)*	(1.89–10.72)*	(1.12–7.08)*	(0.93–2.79)	(0.97–2.77)	(1.34–5.29)*	(1.15–4.40)*	(1.52–5.82)*	(0.81–3.48)	(1.21–5.11)*	(0.53–2.77)

*Note:* ART, antiretroviral therapy. Exploratory multivariable logistic regression models assessed for all independent variables shown in table. Asterisks (*) indicate statistically significant results at *P* < 0.05.

a Respondents classified as reporting any level of suboptimal adherence if they provided a response of ‘Rarely’, ‘Sometimes’, ‘Often’, or ‘Very Often’ to the question: ‘When we consider adherence to treatment, not only in terms of missed doses but also taking the pills at the right time and under the right conditions without overdosing, in the past month how often have you missed taking your HIV pills exactly as prescribed by your HIV physician?’ Each of the specific reasons for missing ART in the table above were dichotomized as 0 or 1. Participants were coded as 1 if they missed ART ‘Sometimes’, ‘Often’, or ‘Very Often’; they were coded as 0 if they reported missing ‘Rarely’ or ‘Never’. Reasons are not mutually exclusive.

b A history of a major side effect was said to be present if the respondent reported a past adverse effect from HIV medication (e.g. ‘stomach/gastric problems because of the medication’ or ‘difficulties taking my HIV treatment as I was having too many side effects’), that led to stopping ART, switching ART, or failing to achieve viral suppression from non-adherence. Difficulty swallowing (i.e. dysphagia) that was elicited by the medicine directly (e.g. size of the pill) and not from an underlying medical condition was also classified as a side effect of the medicine.

c Includes malabsorption, interfering gastrointestinal conditions, and neurocognitive/mental health conditions.

As shown in figure 1, striking similarities were generally seen when comparing reasons perceived by HCPs (*n* = 120) for their patients missing ART ‘Often’ vs. PLWHIV-reported reasons for missing ART among those who reported missing ART ≥ ‘Rarely’ (*n* = 392). Nonetheless, several medical reasons for missing ART were reported higher among PLWHIV than perceived among HCPs. For example, 5.0% of HCPs perceived that their patients missed ART ‘Often’ because of difficulty swallowing whereas 14.8% of PLWHIV who reported missing ART ≥ ‘Rarely’ cited difficulty swallowing as a reason for missing ART. Conversely, several nonmedical reasons for missing ART were estimated to a higher degree by HCPs than reported among PLWHIV. For example, 27.5% of HCPs perceived that their patients missed ART ‘Often’ because of running out of pills whereas only 17.9% of PLWHIV who reported missing ART ≥ ‘Rarely’ reported this factor as a reason for missing ART.

### Perceptions towards long-acting HIV treatment and its role in helping with adherence

In the three countries (France, Germany and the UK) where surveyed PLWHIV were asked whether they were part of a HIV support group or program that helps to remind them how to best take their HIV treatment every day, 14.7% (79/538) affirmed being part of such a program. Similarly, HCPs reported that 15.2% of their patients were enrolled in an adherence support program.

Among PLWHIV who reported missing ART ≥ ‘Rarely’ in all four countries assessed and responded to the question on the novel long-acting regimen, 80.5% (301/374) perceived that the new long-acting treatment would help improve their adherence to ART. Among this population, factors associated with receptivity towards the described long-acting regimen (i.e. belief it would help improve their adherence) included those reporting vs. not reporting dysphagia (AOR = 2.97, 95% CI = 1.26–7.01), gastrointestinal ART side effects (AOR = 2.27, 95% CI = 1.23–4.22) or neurocognitive/mental health conditions (AOR = 1.93, 95% CI = 1.08–3.45). Compared with those with a post-graduate degree, those with a college degree were more receptive towards the described long-acting regimen in terms of perceiving it may help improve their adherence (AOR = 2.65, 95% CI = 1.28–5.46). Older adults aged 50+ years were less receptive that a hypothetical long-acting regimen would help improve their adherence compared with <50-year olds (AOR = 0.51, 95% CI = 0.28–0.94). Gender differences were not statistically significant.

Of all HCPs, 84.2% (101/120) indicated they Definitely or Probably Will Offer a hypothetical long-acting regimen ‘for patients who have suboptimal levels of adherence to daily oral therapy (50–90%) for non-medical reasons’. HCP willingness to offer a long-acting regimen to patients with suboptimal adherence did not vary significantly by the distribution of their patients on ART (*P* = 0.723), virally suppressed (*P* = 0.665), or even perceived adherence levels (*P* = 0.356, Supplementary table S1). The only factor that was significantly associated with HCP willingness to offer was the distribution of patients with dysphagia; HCPs who indicated they probably will offer a long-acting regimen reported the highest percentage of managed patients with dysphagia (an estimated 13.1% of their patients) whereas HCPs indicating that they Definitely or Probably Won’t Offer/Not Sure had the lowest percentage of patients with dysphagia (an estimated 6.4% of their patients) (Supplementary table S1).

## Discussion

We found that about a quarter of PLWHIV (23.8%) reported suboptimal adherence to HIV treatment. Emotional challenges and a busy lifestyle were the leading reasons for missing ART. Consistent with previous research,^24^^,^^25^ the likelihood of reporting suboptimal adherence in our study was higher among women, those with comorbid conditions that made oral intake challenging, those with privacy concerns, or stressed by their ART routine, and those with longer duration of HIV. Women are more likely to be caregivers, and to face some additional, unique challenges that may interfere with their adherence.^26–28^ The higher reporting of suboptimal adherence among those with other medical conditions may be multifactorial; for example, because of eating-induced pain with gastric ulcers,^29^ affected individuals may fail to take their HIV medication at the right time, or under the right conditions, especially if there are food requirements with the medication. HIV-associated neurocognitive disorders (HAND) may contribute to suboptimal adherence because of memory deficits.^30^ The higher reporting of suboptimal adherence among those with longer duration of disease may partly be explained by the observation that increasing duration of HIV may be associated with higher burden of treatment-related challenges like insomnia, substance use, polypharmacy, and mental illness, which may impact adherence.^24^^,^^31^^,^^32^

Interventions to improve adherence among PLWHIV from the literature have included behavioral/educational, pharmacists-led, reminders, and regimen simplification.^33^ Previous research show mixed results regarding the effectiveness of behavioral interventions in improving adherence, especially among the most vulnerable patient groups.^34^ Regimen simplification has been demonstrated to have a significant impact in achieving optimal adherence as complex regimens may compromise QoL, and are challenging to follow exactly as prescribed.^19^^,^^20^

Whereas only 5% of HCPs perceived that their patients missed ART ‘Often’ because of difficulty swallowing, 14.8% of PLWHIV with any level of suboptimal adherence cited difficulty swallowing as a reason for missing ART. This could indicate the need for more thorough evaluation of patients’ medical needs by physicians. Given that drug effects and their side effects can change over time, it is critical for PLWHIV and their HCPs to routinely discuss side effects which the patient may be experiencing. Such patient-centered care can improve treatment satisfaction, and in turn, adherence to ART.^35^

Most HCPs (84.2%) were willing to offer a long-acting regimen ‘for patients who have suboptimal levels of adherence to daily oral therapy for non-medical reasons (e.g. travels, age/maturity, work, recreational drug use)’ and this was independent of HCPs’ perceived level of adherence among their patients. Indeed, HCPs’ perception of patients’ adherence levels, even if based directly on patient reports, may be unreliable, partly because of social desirability bias on the part of patients or forgetfulness, especially if failure to take the medication as prescribed is unintentional.^36^ Failure to dose at the right time was the most common form of suboptimal adherence in our study, followed by missing a dose altogether. Novel HIV treatments that are designed to be administered every 2 months at points of care by health professionals could help to address several of these challenges. Because CAB + RPV LA is directly observed therapy, this removes the need for adherence to daily dosing,^17^^,^^18^ allowing HCPs to have more certainty about the protection of their patients. Such a regimen could also allow for more accurate tracking of doses received by the patient, thus overcoming the inherent subjectivity in assessing and measuring adherence with self-reported information. Furthermore, the fact that these new treatments will be administered only six times in a year (vs. 365 times for daily oral regimens) could greatly improve QoL among PLWHIV.^36^^,^^37^ Of PLWHIV who reported missing ART ≥ ‘Rarely’, most (80.5%) believed that the described long-acting regimen would help improve their level of adherence, especially those with a medical condition that made oral administration challenging, those with confidentiality concerns, or with treatment-related emotional challenges.

The strengths of this study include multi-country analyses with remarkable alignment between countries. Examining this issue of adherence from the complementary perspectives of PLWHIV and HCPs provides a more complete picture to help address unmet needs. However, this study has some limitations. First, the cross-sectional design and self-reported measures preclude any causal inferences, and only associations can be drawn. Second, it is possible that the surveyed PLWHIV may be receiving care from different HCPs than those recruited in our study; this may explain some of the discrepancy in the estimates reported by PLWHIV vs. HCPs in our study. Also, there may have been measurement bias (e.g. misreporting) from poor recall and social desirability bias. There is also potential for selection bias (e.g. non-coverage or non-response bias) because of the non-probabilistic sampling. We however tried to minimize these biases by ensuring that the distribution of the sampled population matched the national distribution of PLWHIV in each of the four countries during the sampling/recruitment phase. That this was effective is reflected in the observation that results from self-weighted analyses presented in this article were remarkably close to those weighted for age, gender, sexual orientation, and nativity status (data not shown). For example, among all PLWHIV on ART, prevalence of suboptimal adherence in general was 23.8% in unweighted analyses, vs. 23.0% in weighted analyses. Unweighted vs. weighted results by country were as follows: France (27.1% vs. 24.7%); Germany (21.7% vs. 20.0%); Italy (20.7% vs. 21.4%); and the UK (26.0% vs. 25.9%). These data are suggestive that the magnitude of selection bias is small.

## Conclusion

Overall, about 1 in 4 of surveyed PLWHIV (23.8%) reported suboptimal adherence to treatment; surveyed physicians estimated that 33.6% of their patients had suboptimal adherence. Surveyed PLWHIV with medical, psychosocial and emotional challenges related to treatment reported poorer adherence, including those with medical conditions that made oral intake challenging, those with privacy concerns (hiding medications), or those stressed by their daily oral ART routine. PLWHIV and HCP perceived that long-acting regimens could help with suboptimal adherence; about 4 in 5 (80.5%) of PLWHIV who reported any level of suboptimal adherence perceived that long-acting regimens would help improve their adherence to ART. Likewise, over 4 in 5 (84.2%) of surveyed HCPs were willing to offer long-acting regimens to patients with suboptimal adherence. Providing patients with alternative regimens to daily oral ART, such as long-acting regimens, could improve treatment outcomes, including QoL and adherence.

## Supplementary data

Supplementary data are available at *EURPUB* online.

## Supplementary Material

ckaa229_Supplementary_Data
