# Supplementary material for: Dose-related and contextual aspects of suboptimal adherence to antiretroviral therapy among persons living with HIV in Western Europe
Source: Eur J Public Health. 2021 Jan 18;31(3):567–75. doi: 10.1093/eurpub/ckaa229 (PMC8277220; doi:10.1093/eurpub/ckaa229)
Supplement: ckaa229_Supplementary_Data [file ckaa229_supplementary_data.docx]

Supplemental Table 1. Distribution of patients managed by HCPs on various clinical characteristics, by their willingness to offer a hypothetical long-acting regimen for suboptimal adherence as well as percentage of PLWHIV with some level of suboptimal adherence ^a^, by medical condition

|  | Indicator | Prevalence of outcome, % | p-value (χ^2^ test) |
| --- | --- | --- | --- |
| **HCP reported distribution of their patients, by willingness to offer a hypothetical long-acting regimen** | **Managed patients on ART**, by HCP willingness to offer a hypothetical long-acting regimen "for patients who have suboptimal levels of adherence to daily oral therapy (50-90%) for non-medical reasons" |  | 0.723 |
|  | Definitely Will Offer (51) | 86.4 |  |
|  | Probably Will Offer (50) | 86.0 |  |
|  | Definitely or Probably Won't Offer/Not Sure (19) | 83.0 |  |
|  | **Managed patients with viral suppression**, by HCP willingness to offer a hypothetical long-acting regimen "for patients who have suboptimal levels of adherence to daily oral therapy (50-90%) for non-medical reasons" |  | 0.665 |
|  | Definitely Will Offer (51) | 85.1 |  |
|  | Probably Will Offer (50) | 84.0 |  |
|  | Definitely or Probably Won't Offer/Not Sure (19) | 80.4 |  |
|  | **Managed patients with suboptimal adherence**, by HCP willingness to offer a hypothetical long-acting regimen "for patients who have suboptimal levels of adherence to daily oral therapy (50-90%) for non-medical reasons" |  | 0.356 |
|  | Definitely Will Offer (51) | 36.6 |  |
|  | Probably Will Offer (50) | 29.0 |  |
|  | Definitely or Probably Won't Offer/Not Sure (19) | 36.9 |  |
|  | **Managed patients with CNS conditions**, by HCP willingness to offer a hypothetical long-acting regimen "for patients who have suboptimal levels of adherence to daily oral therapy (50-90%) for non-medical reasons" |  | 0.108 |
|  | Definitely Will Offer (51) | 12.1 |  |
|  | Probably Will Offer (50) | 13.3 |  |
|  | Definitely or Probably Won't Offer/Not Sure (19) | 5.9 |  |
|  | **Managed patients with interfering gastrointestinal conditions**, by HCP willingness to offer a hypothetical long-acting regimen "for patients who have suboptimal levels of adherence to daily oral therapy (50-90%) for non-medical reasons" |  | 0.258 |
|  | Definitely Will Offer (51) | 8.4 |  |
|  | Probably Will Offer (50) | 12.2 |  |
|  | Definitely or Probably Won't Offer/Not Sure (19) | 11.1 |  |
|  | **Managed patients with malabsorption**, by HCP willingness to offer a hypothetical long-acting regimen "for patients who have suboptimal levels of adherence to daily oral therapy (50-90%) for non-medical reasons" |  | 0.203 |
|  | Definitely Will Offer (51) | 7.8 |  |
|  | Probably Will Offer (50) | 12.3 |  |
|  | Definitely or Probably Won't Offer/Not Sure (19) | 8.3 |  |
|  | **Managed patients with dysphagia**, by HCP willingness to offer a hypothetical long-acting regimen "for patients who have suboptimal levels of adherence to daily oral therapy (50-90%) for non-medical reasons" |  | 0.043 |
|  | Definitely Will Offer (51) | 7.6 |  |
|  | Probably Will Offer (50) | 13.1 |  |
|  | Definitely or Probably Won't Offer/Not Sure (19) | 6.4 |  |
| **Percentage of PLWHIV reporting some level of suboptimal adherence, by medical condition reported** | Prevalence of some level of suboptimal adherence, **by self-reported diagnosis of malabsorption** |  | 0.386 |
|  | Malabsorption not reported (632) | 23.4 |  |
|  | Malabsorption reported (56) | 28.6 |  |
|  | Prevalence of some level of suboptimal adherence, **by self-reported diagnosis of dysphagia** |  | <0.001 |
|  | Dysphagia not reported (565) | 18.9 |  |
|  | Dysphagia reported (123) | 46.3 |  |
|  | Prevalence of some level of suboptimal adherence, **by self-reported diagnosis of Interfering gastrointestinal conditions** |  | 0.085 |
|  | Interfering gastrointestinal conditions not reported (560) | 22.5 |  |
|  | Interfering gastrointestinal conditions reported (128) | 29.7 |  |
|  | Prevalence of some level of suboptimal adherence, **by self-reported diagnosis of CNS conditions** |  | <0.001 |
|  | Neurocognitive/mental health conditions not reported (444) | 19.1 |  |
|  | Neurocognitive/mental health conditions reported (244) | 32.4 |  |

Note: HCP = Healthcare provider; PLWHIV = People living with HIV

^a^ Respondents were classified as reporting some level of suboptimal adherence if they provided a response of “Sometimes”, “Often”, or “Very Often” to the question: “When we consider adherence to treatment, not only in terms of missed doses but also taking the pills at the right time and under the right conditions without overdosing, in the past month how often have you missed taking your HIV pills exactly as prescribed by your HIV physician?”
